# Dorsal agenesis of the pancreas: an incidental finding

**DOI:** 10.1093/jscr/rjae655

**Published:** 2024-10-17

**Authors:** Daphne Cauchi, Shaun Mangion, Noel Cassar

**Affiliations:** Department of Surgery, Mater Dei Hospital, Triq Dun Karm, Msida MSD 2090, Malta; Department of Surgery, Mater Dei Hospital, Triq Dun Karm, Msida MSD 2090, Malta; Department of Surgery, Mater Dei Hospital, Triq Dun Karm, Msida MSD 2090, Malta

**Keywords:** agenesis of dorsal pancreas, case report, pancreatic dysplasia

## Abstract

This case report focuses on a 29-year-old female who presented with acute abdominal pain at Mater Dei Hospital, Malta. Her clinical presentation, followed up by diagnostic imaging, led to the diagnosis of a rare congenital abnormality known as dorsal agenesis of the pancreas. This condition is characterized by the absence or underdevelopment of the dorsal portion of the pancreas, a crucial aspect of pancreatic anatomy and function. The following text details the clinical presentation, diagnostic findings, and the broader implications of dorsal pancreatic agenesis in medical practice, reflecting on the rarity of this condition and the complexity of its diagnosis and management.

## Introduction

Dorsal agenesis of the pancreas is a rare developmental abnormality that can be partial or complete in nature. Since its initial documentation in 1911, only a few cases have been documented in published literature [1]. Below we report a case of agenesis of the dorsal pancreas, which was discovered following investigation for presentation of abdominal pain at Accident and Emergency (A&E).

## Case report

**Figure 1:**
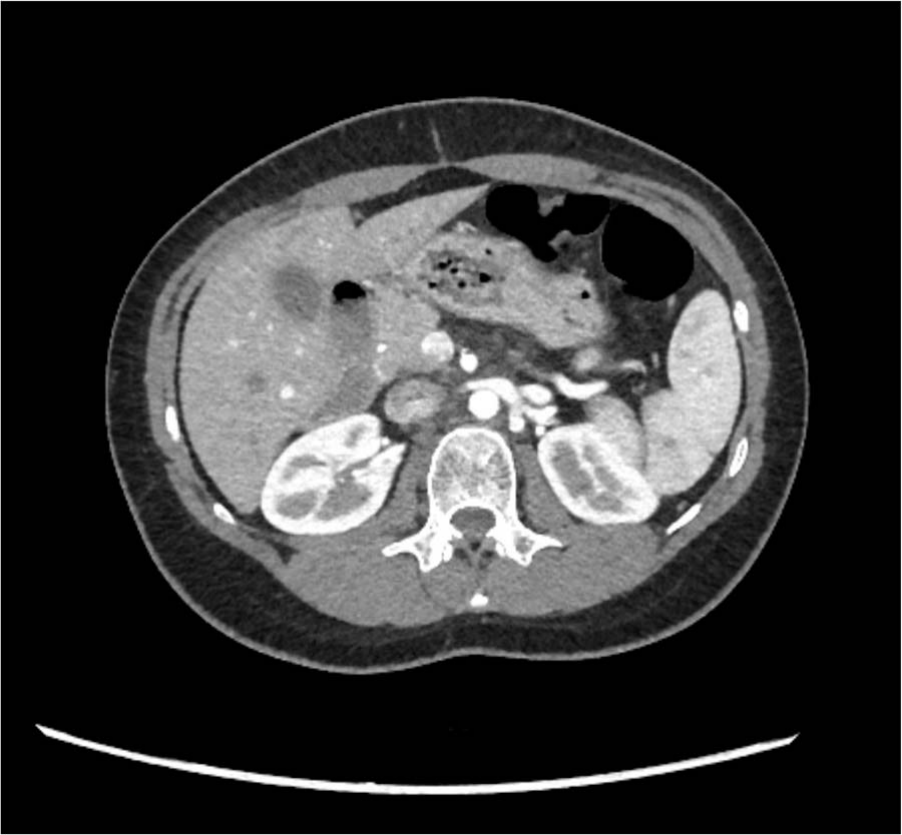
CT pancreas demonstrating absent body and tail of pancreas in axial view.

**Figure 2:**
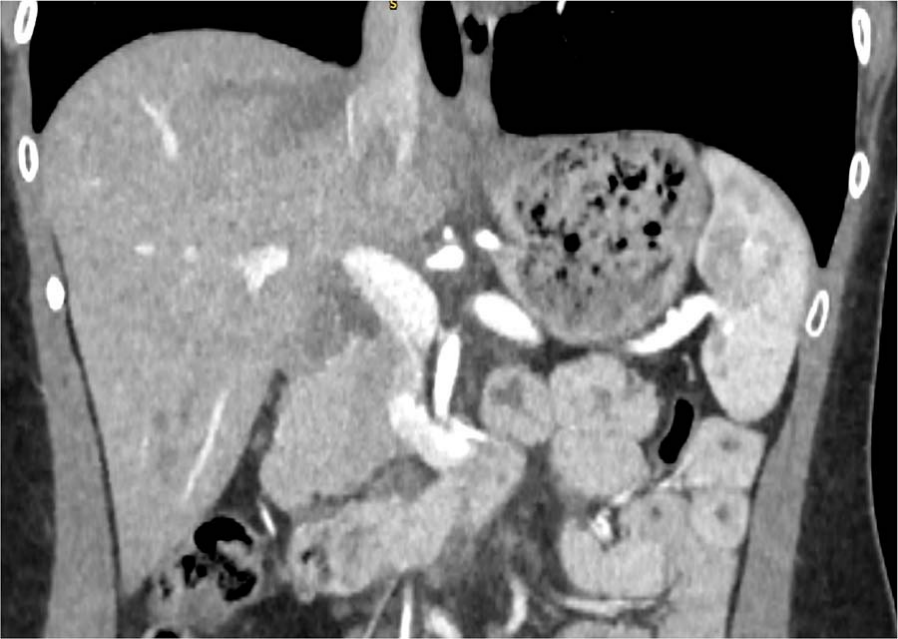
CT pancreas demonstrating absent body and tail of pancreas in coronal view.

A 29-year-old female, with a past medical history of asthma and anxiety, presented to A&E Department at Mater Dei Hospital in view of a 2-day history of worsening right-sided colicky flank pain that radiated down to the right iliac fossa. Pain relief, including paracetamol and NSAIDS, alleviated her symptoms temporarily; however, pain recurred after a few hours. At A&E, patient’s parameters were noted to be stable throughout the review. On examination, the patient’s abdomen was noted to be soft with tenderness over the right flank. No guarding was noted, and renal punch was negative bilaterally. Blood investigations were all within normal limits, and urinalysis was normal. In view of the examination findings, a computed tomography (CT) of the kidney, ureters, and bladder was ordered, which noted an enlarged pancreatic head measuring 3 cm in diameter with noted upstream atrophic changes in the body and tail with fat stranding; prominent mesenteric lymph nodes were also noted. Based on the previous CT findings, a contrast CT of the pancreas was ordered, which revealed that the body and tail of the pancreas were absent; however, the pancreatic head was noted to be enlarged. This can be seen in Figs 1 and 2. Prominent lymph nodes with ground glass changes in the mesenteric fat were noted. CT pancreas therefore confirmed the diagnosis of dorsal agenesis of the pancreas. The patient was discharged home on pain relief and an outpatient’s appointment with the hepatobiliary team. Upon review at outpatients, the patient was well. She noted that she had been complaining of intermittent right loin pain over the past 3 years. Over the 2 months from review at A&E, the patient was well and denied further abdominal pain. In view of this, the patient was reassured and discharged. She was advised to seek medical advice should symptoms recur.

## Discussion

Agenesis of the dorsal pancreas is a rare congenital abnormality that was first documented in 1911 as an autopsy finding. In the century since its documentation, <60 cases have been documented, according to a recent paper by Ehrhardt and Gomez [1].

The pancreas develops from two primitive endodermal tissues during the fifth week of gestation. The two primitive endodermal tissues are referred to as the ventral and dorsal bud. The ventral bud, which is considerably smaller than the dorsal bud, is found between the duodenum and common bile duct, while the dorsal bud is found higher in comparison to the ventral bud and develops toward the spine, finally settling in the laminas of the mesentery of the duodenum and stomach. As the hepatic diverticulum and foregut tube grow, counterclockwise rotation of the ventral bud causes a shift into the dorsal direction. Development finishes off with the ventral bud being found under and behind the dorsal bud. The ventral bud goes on to form the inferior part of the head of the pancreas and uncinate process, while the dorsal bud will then form the superior part of the head of the pancreas, body, and tail [2]. At the seventh week of gestation, the ventral and dorsal pancreatic buds fuse to form the pancreas while also connecting to the common bile duct and duodenum. The dorsal bud forms a microscopic luminal network, which will go on to form the acinar and ductal systems, which will eventually responsible for the exocrine secretions. The dorsal bud is also associated with the development of the accessory duct of Santorini, while the ventral duct is associated with the main pancreatic duct of Wirsung [1].

Dorsal pancreatic agenesis is associated with three main gene mutations. The first gene to be reportedly associated with dorsal agenesis of the pancreas was a frameshift point mutation in pancreatic and duodenal homeobox 1 (PDX1) found in the pancreatic endodermal buds. A defect in this gene will result in insulin requiring hyperglycemia secondary to exocrine pancreatic insufficiency. A nonsense mutation involving transcription factor 1A (PTF1A) was the second gene associated with pancreatic agenesis as well as cerebellar agenesis. Finally, an insufficiency in the GATA-binding protein 6 (GATA6) was the latest genetic defect linked to pancreatic agenesis. Of note, the GATA6 gene mutation has also been associated with pancreatic hypoplasia and other structural cardiac defects [3].

Agenesis of the dorsal pancreas can occur either partially or completely. In cases of complete agenesis, the body and tail of the pancreas, along with the minor papilla and the accessory pancreatic duct, are missing. In partial agenesis, however, varying amounts of the pancreatic body and tail remain, accompanied by the minor papilla and a portion of the accessory pancreatic duct [4].

Dorsal agenesis of the pancreas can remain asymptomatic, and detection may be following investigation for unrelated conditions; however, it may also present with abdominal pain and bloating, which may be related to diabetes mellitus, resulting in autonomic neuropathy causing dysfunction of the sphincter of Oddi [5]. This noted dysfunction in the sphincter of Oddi may also be linked with acute pancreatitis, pancreatic duct hypertension, increased secretion of pancreatic juice, and compensatory pancreatic head hypertrophy [6]. ADP has been linked to pancreatic neoplasia, such as cystic lesions, malignant intraductal papillary mucinous neoplasia (IPMN), and adenocarcinoma, which some studies have attributed to chronic pancreatitis [5, 7]. Other presentations of ADP may be by an abdominal mass, bile duct obstruction, duodenal obstruction, or polysplenia [8].

Previously, diagnosis of ADP was on autopsy or laparotomy; however, advancing medical imaging, including the use of ultrasound, CT, magnetic resonance cholangiopancreatography (MRCP), and endoscopic retrograde cholangiopancreatography (ERCP), has helped identify this congenital abnormality. Ultrasonography, although fast and readily available, fails to identify this ADP, as bowel gas may obscure image acquisition [9]. CT can detect the pancreatic head; however, MRCP or ERCP is able to visualize the pancreatic ductal anatomy adequately and therefore confirm the diagnosis of partial or complete ADP [10].

## Conclusion

Agenesis of the dorsal pancreas is an exceedingly rare congenital abnormality with unclear pathogenesis, which should be considered when the pancreatic body and tail are not visualized on routine imaging. As it is an uncommon finding, management will depend upon the presentation of symptoms.
